# Molecular portraits: the evolution of the concept of transcriptome-based cancer signatures

**DOI:** 10.1093/bib/bbv013

**Published:** 2015-03-31

**Authors:** Angelika Modelska, Alessandro Quattrone, Angela Re

**Keywords:** transcriptomes, signatures, cancer, heterogeneity, omics

## Abstract

Cancer results from dysregulation of multiple steps of gene expression programs. We review how transcriptome profiling has been widely explored for cancer classification and biomarker discovery but resulted in limited clinical impact. Therefore, we discuss alternative and complementary omics approaches.

## Introduction

**Figure 1.**
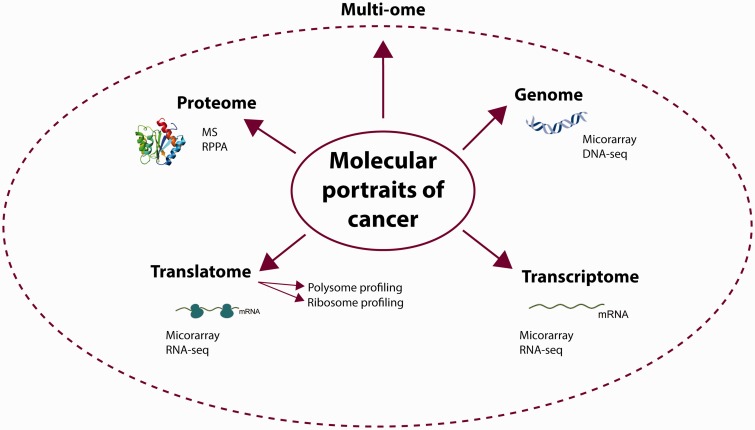
Obtaining molecular portraits of cancer. Different omics fields are listed together with the main methods used to obtain results. DNA-seq—DNA sequencing; RNA-seq—RNA sequencing.

Each step of the gene expression program is tightly controlled by regulatory circuits [1]. Cancer is a disease resulting from dysregulation of these circuits, which can occur at any point of the control [2, 3]. Hence, each of the ‘molecular portraits’ of cancer obtained through high-throughput profiling (omics) technologies (Figure 1) bears fingerprints, which in principle can be exploited to identify biomarker genes for diagnosis and prognosis, and to find suitable therapeutic targets.

**Figure 2.**
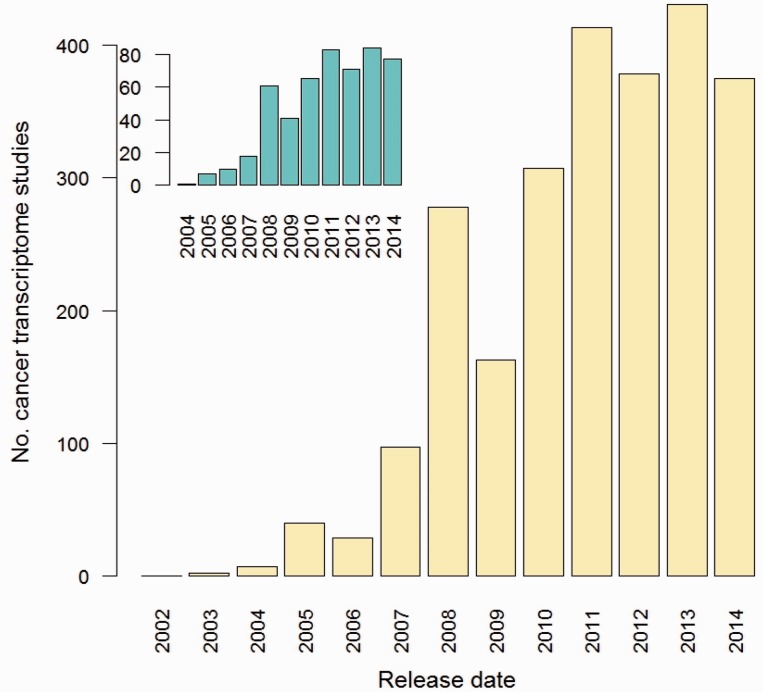
The increase in the number of cancer transcriptome profiling studies. ArrayExpress was queried for human cancer data sets acquired from at least 30 samples by RNA microarray or sequencing assays. Main bar plot:data sets acquired from any cancer type; insert plot: data sets from breast cancer studies only.

In the nearly 15 years from when the first cancer portrait was painted as an early outcome of the microarray technology [4], the great majority of the subsequent analyses have been limited to the first two levels of gene expression, the linear DNA sequence and the steady-state levels of mRNAs (i.e. genome and transcriptome profiling). Despite continuous advances in the affordability of microarrays to quantify copy number variations and mRNA levels, the transition to the massively parallel sequencing technology, which has an improved resolution, a greater dynamic range and an increased sensitivity depending on the depth of sequencing [5–7], has rendered sequencing-based cancer portraits definitely more informative than those relying on hybridization to probes. In the meanwhile, public repositories of high-throughput analysis of biological data have been heavily loaded with profiles of virtually every cancer type, following a rapid pace of accumulation (Figure 2) also owing to multisite and international projects such as TGCA [8] and ICGP [9]. As a result, our understanding of the anatomy of cancer genomes and transcriptomes has substantially improved. We now map precisely point mutations, copy number aberrations, translocations, splicing defects, alterations of transcription start or end and differential expression [2, 7].

Looking retrospectively, despite this increased mapping ability of genomic and transcriptomic aberrations, the usefulness of omics assay in the distinct tasks of classifying tumor subtypes, predicting clinical outcome (clinical validity) as well as treatment response (clinical utility) [10] appears rather unsatisfactory. At the basis of the poor clinical usefulness of the molecular portraits produced until now could lie the inability to account for the biological complexity of cancer [11, 12]. It is still out of reach to comprehend in an informative way the multiplicity of processes in the path from DNA to protein, which are known to be dysregulated [13], and the underlying intra-tumor variation poses great difficulties [14–16]. Moreover, tumors bear both ‘driver’ and ‘passenger’ mutations that are difficult to distinguish [17], and are characterized by the tendency to undergo a Darwinian evolution and to evolve with time and treatment [18–20]. Admittedly, the major challenge in cancer research is the heterogeneity of this disease, not only between different tissues and cancer types, but also within the same cancer type among the different patients and temporally in the same patient. Additional hurdles related to technical approaches include the analysis of complex biological materials, insufficient sample size and improper composition of study cohorts, lack of clear objectives and thoughtful statistical design upfront of the experiments and an improper subsequent data analysis including the application of arbitrary thresholds [21, 22]. Finally, to understand highly interconnected mechanisms that drive cancer and to impact on oncology practice, we have to evaluate the still largely unknown potential of protein levels and protein posttranslational modifications, as well as integrative views of molecular mechanisms at multiple regulatory levels.

In this review, we examine the possible reasons of the failure to substantially improve clinical practice using the currently available, predominantly transcriptome-based molecular portraits, and identify recent advancements that could lead to more success in the future as well as present current limitations in the field.

## Transcriptome-based portraits: the case of breast cancer

Breast cancer is heterogeneous, consisting of cases that are substantially different in their molecular and clinical characteristics. Owing to this inherent diversity, attempts at class discovery, prediction and management of breast cancer patients pose significant challenges [23]. Breast cancer heterogeneity also prevents the establishment of a multistep model of carcinogenesis, which has been possible in other cancer types, such as colorectal or pancreatic cancer [24, 25]. Despite such complexity, a nearly 15 years long examination of breast cancer transcriptome profiles resulted in substantial contributions into subtyping and assessment of the prognostic (providing information on the likely outcome of the cancer disease in an untreated individual) and predictive (providing information on the likely benefit from treatment) value of gene signatures. Therefore, we use this tumor type as a paradigm of how transcriptome profiles have contributed to the clinical management of this disease.

The initial molecular classification of breast cancer was based on transcriptome profiling and resulted in five subgroups, namely, Luminal A, Luminal B, Basal-like, Normal-like and HER-2 positive, which were subsequently proven to be associated with distinct clinical outcomes [4, 26]. Following the discovery of the initial subgroups, further subtyping has been proposed by several studies. A meta-analysis approach on transcriptome signatures derived from 42 breast cancer gene expression studies yielded a set of 117 genes that were common to 12–36% of the studies [27]. The low number of genes common to the meta-analyzed breast cancer signatures did not prevent the meta-signature from improving breast cancer patient stratification. The difficulty of developing transcriptome-based models in breast cancer was also recently typified by a study that, by showing that most random gene lists associate with patient prognosis, questioned the implicit assignment of biological significance to the variables found in association with patient prognosis [28]. Such justified critiques are helping the research community to evolve improved analytical methodologies to identify biologically informative cancer prognostic signatures [29].

One of the biggest hurdles for researchers in this field is the sample size that needs to be increased to obtain meaningful results for such a complex disease as breast cancer. The recent METABRIC (Molecular Taxonomy of Breast Cancer International Consortium) study, using 2000 breast cancer patients with long-term clinical outcome information, is the latest wide-scale effort in producing a molecular classification of the tumor [30]. Therein, integrated analysis of genome and transcriptome portraits resulted in the robust identification of 10 subgroups, each being reproducible in the validation cohort and thus representing trustable tumor subtypes. METABRIC findings may help the researchers to better define future studies and plan clinical trials, as each subtype is likely to need a different treatment strategy to overcome the disease [23]. Furthermore, an assessment of the clinical validity of the integrative subtypes derived from METABRIC has been undertaken [31], even if it is worth noting that the METABRIC subtypes were solely conceived with the intention of best representing breast tumor biology and identifying potential molecular drivers. In general, the clinical potential of breast tumor subtyping warrants further study. At the moment, the descriptive power to define breast cancer subtype is being gained with limited translation into the clinic.

Out of the vast number of proposed breast cancer signatures, only a few of them have been licensed for use in the clinic, the most prominent ones listed in Table 1. The evidence base for these assays develops primarily through observational studies of limited sample sizes in diverse settings. As a matter of fact, unmet minimum sample size of study cohorts hampers the assessment of statistical performances, and prediction strength is known to vary considerably depending on which patient populations and what end point the assay was optimized for. Nonetheless, major conclusions were conveyed by previous systematic reviews and meta-analyses [37]. Evidence of analytic validity (technical performance characteristics) and reproducibility was found limited for all assays. Overall, these assays showed clinical validity and utility, albeit to a different extent. OncotypeDX, in particular, positions at the most advanced point in the development pathway, being the only assay that can predict treatment benefit [38, 39]. Recently, the Oncotype DX assay was modified to predict local recurrence risk and to guide individualized selection of treatment after surgical excision for women with newly diagnosed ductal carcinoma *in situ* of the breast who meet the ECOG E5194 criteria [40]. The cost-effectiveness of the assays remains inconclusive [41]. For all tests, the relationship of predicted to observed risk in different populations and their added value over conventional predictors, optimal implementation and relevance to patients receiving current therapies need further study, in particular in large cohorts. These should prove key to understanding whether we can rely on these assays rather than on standard clinicopathological features for treatment decisions [42, 43]. For now, they remain only complementary to the traditional tests. A comparative study of gene signatures showed that predictions of distinct assays were similar despite the limited overlap between the genes underlying the assays [44]. It is worth noting that observing a low overlap across gene signatures is not relevant from a purely clinical point of view because it can be due to the presence of multiple genes with similar moderate correlation to the clinical outcome. This scenario was highlighted in a study where different lists of survival prognostic biomarkers were generated from a single breast cancer data set [45].

**Table 1.** Most prominent signatures used in breast cancer clinical practice

Assay name (producer)	Description	Number of genes assayed	Reference
MammaPrint (Agedia)	Assesses the risk of metastasis of early-stage breast cancer, and whether a patient will benefit from chemotherapy.	70	[32]
Oncotype DX (Genomic Health)	Assesses the chances of disease recurrence in women with early-stage estrogen receptor positive breast cancer and the benefit from certain types of chemotherapy.	21	[33]
MapQuant Dx (Ipsogen)	Assesses the histological grade and thus predicts the benefit from chemotherapy.	97	[34, 35]
THEROS CancerTYPE ID (Biotheranostics)	Identifies the origin of cancers with unknown or uncertain primary site.	92	[36]

Difficulties to acquire robust cancer subtypes as well prognostic and predictive biomarkers could depend on the limitations of transcriptome-based profiling as the technology of choice. One of the reasons could be that transcriptome-based molecular profiles are not true portraits of the diseased state but mere shadows, as mRNA and protein levels tend to be rather poorly correlated (see below) and depend on genomic lesions in a complex way.

## The discordance of information contained in different molecular portraits

The expression of many genes predicted to drive cancer phenotypes is weakly correlated with their copy number, and protein levels are poorly predicted by transcript levels, both in tumor samples and cell lines [46–48]. As the proteome is believed to more closely represent the physiological state of the cell than the genome or the transcriptome, the discordance between transcriptome and proteome data should draw attention to the limits of many cancer studies that use transcriptome as a surrogate for proteome to reconstruct molecular portraits and to identify cancer signatures.

The low estimates of correlation between transcriptome and proteome data could be partially owing to technical reasons, such as different scalability between platforms, detection bias, different kinetics of synthesis and degradation between transcripts and proteins or side effects resulting from the use of drugs that inhibit transcription and translation [49, 50]. In addition, the partial correlation between transcript and protein levels was not mitigated when the quantification was addressed in optimal experimental conditions on a genome-wide scale in unperturbed mammalian cells [51]. In this study, transcript levels could explain only 40% of the variance in protein levels, which, albeit higher than in previous reports [49, 50], is limited. An estimate of the correlation between transcript and protein levels was also addressed computationally. Combining steady-state mRNA and protein levels with the analysis of the sequence features related to translation and protein degradation, allowed to demonstrate that the transcript levels and structural features together could explain two-thirds of the variation in protein levels, whereas transcript levels alone could explain only 25–30% of it [52].

The relative importance of variation in the transcriptome and the translatome (the latter being a subfraction of the transcriptome composed of mRNAs engaged in translation) on treatment with different stimuli was assessed in another study [53], which showed an extensive uncoupling of mRNA transcriptome and translatome fluctuations, with up to 90% of significant changes being limited to the translatome. These results, together with others [54, 55], support the notion that the machineries responsible for regulating mRNA levels as well as mRNA translation can remain largely independent of each other. In line with this view, protein levels are more conserved during evolution than the corresponding mRNA levels [56], as assessed looking at seven different species and identifying a selective pressure to maintain protein abundances during evolution even when mRNA abundances diverge. Taken together, the currently available evidence suggests that the process of translation and the abundance of proteins might provide better molecular portraits of healthy and disease states than the transcriptome.

## The use of translatome profiling

Cancer characterization and biomarker development could now take advantage from advances in translatome profiling, which examines translation by characterizing mRNA transcripts engaged in interaction with active ribosomes. Polysome profiling, relying on the analysis of polysomal mRNAs that are first fractionated by sucrose gradient centrifugation, is commonly used to assess the translational efficiency of transcripts. To date, no studies have been conducted to assess the utility of the translatome in the clinical context, with only one report on polysome profiling from solid tissues [57]. More recently, ribosome profiling data have also been proposed as a proxy for protein abundance [58, 59], and this method is proving useful to evaluate translation deregulation in diseased states [60–62]. Nevertheless, the broad application of polysome and ribosome profiling is limited by the lack of high-throughput settings of the procedures. All in all, translation profiling techniques can be helpful to uncover cancer cell behaviors specifically steered by disarranged translation, and therefore to suggest novel therapeutic targets. However, this field certainly needs further advancements and better standardization of protocols to fulfill its full promise in the clinics.

## The use of proteome profiling

The ability to understand proteomes brings us a step closer to the physiological state of a cell, and therefore proteomics promises to provide enhanced ability to predict the disease course, to promote insights in cancer deregulated pathways and associated mechanisms of resistance to treatment. Proteomics has now advanced sufficiently to allow us to observe the variation of thousands of proteins [63]. However, proteome determination still lags behind transcriptome assays in terms of sensitivity, accuracy and scalability. Proteomics-based cancer studies are inherently more complicated, owing to large dynamic range of concentrations of molecular species and the absence of means to amplify them, low relative abundance of many biomarkers and the extent of protein variability owning to splicing and protein modifications [64]. Recent advances in technology platforms relying on mass spectrometry (MS), sample handling and bioinformatics solutions for data analysis have delivered high-coverage surveys of the proteome [65]. Over 4 years, a 100-fold increase in throughput has been achieved [66, 67], which brings the analysis of complete proteomes within reach. Several initiatives, such as the Human Proteome Map, ProteomicsDB, the Chromosome-Centric Human Proteome Project, have demonstrated the feasibility of complementing available genome and transcriptome normal data by comprehensively exploring the proteome on progressively larger scale in the near future [68–70]. In addition, the Cancer Proteome Atlas (TCPA) provides ready access to cancer proteome data, which were generated using 3467 TCGA tumors and 439 cell line samples by using reverse-phase protein arrays [71]. As a proof of concept, a preliminary analysis of TCPA data has recently proved the association of several proteins with clinical outcome [72], but the limitation of this assay is that it targets only 181 proteins [71]. Furthermore, the Clinical Proteomic Tumor Analysis Consortium, launched by the National Cancer Institute, has released a public repository of well-characterized, MS-based, targeted proteomic assays, including a growing number of cancer types [73].

The proteomes of human blood serum and plasma contain a vast amount of useful information about the cancer state. Blood collection is simple and minimally invasive, making it a medium of choice for diagnostic tests. Until recently, the exploitation of blood proteome for discovery of protein biomarkers has been challenging because of the large number of unique proteins, their degradation products and the broad range of protein concentrations. Nonetheless, advances in proteomic technologies are possibly turning the serum proteome diagnostics into a clinical reality [74, 75]. For instance, the VeriStrat serum proteomic test status was found predictive of differential benefit in terms of overall survival for non-small-cell lung cancer patients treated with second-line erlotinib versus chemotherapy [76].

Proteome-based cancer portraits unfortunately suffer from some pitfalls that are specific to proteomic approaches. Typical challenges in the proteomics workflow include inconsistent proteolysis of samples and protease bias [77], the limited working concentration range of species detectable by the instrumentation, the need for reduction in sample complexity, which otherwise is not directly compatible with MS analysis, and the error-prone association between spectral data, peptides and proteins [78]. Proteomics data interpretation could benefit from evolving methods to model experiment-specific peptide detectability trends, to enable comparative evaluation of protein identification methods and to standardize and expand database annotations that are crucial, being the source used for MS spectra matching. Thus far, a small number of studies performed suitable validation of protein biomarkers derived by proteomics, i.e. in clinical samples with patients’ follow-up data [79–81]. Although proteomics holds many promises, considerable work still lies ahead to accelerate the progress in distinguishing between cancer types in the clinic as well as in defining suitable treatment regimens.

## The use of multi-omic approaches

Improvements in the accuracy and precision of high-throughput technologies have set the stage for the integration of multiple data types. In addition to the mostly investigated transcriptomes and proteomes, additional molecular profiles have been shown informative; for instance, metabolic profiles have an important theoretical advantage in that they are sensitive to both genetic and environmental influences, and can be translated into clinical tools for application to personalized medicine [82–84]. The advantages of integrative analyses of multiple assays are arguably manifold: more fundamental understanding of the complex biology of cancer in its multiple and interrelated facets, power and robustness in tumor classification and prediction of outcome, which cannot be reached by each assay in isolation. A drawback of the integrative methods is a considerable dimensionality issue owing to the increasing of the number of variables without increasing the number of samples. However, an array of techniques are amenable to integrative analysis, such as the preliminary identification of principle components, which are defined as latent axes that maximally capture the variance in the data set, or the imposition of variable sparsity through LASSO constraints [85] or elastic net constraints [86]. To enable the access to multi-assay data and the exchange of integrative analysis methods, storage and informatics platforms such as Rembrandt [87], Synapse [88] and IntOGen [89] have been already established as public resources for open data-driven collaborative research. The methodological choice of the computational analysis depends on the objectives set by the integration of multi-assay data [90, 91]. For example, a number of approaches used different technologies to confirm the findings based on one assay with an additional one [92, 93]. Tumor stratification has been usually addressed by applying clustering-based approaches to multi-assay data types. The most common approach to subtype discovery across multiple data types is to sequentially cluster each type and finally to integrate the results. For instance, integrating six assays by this approach demonstrated the ability to molecularly define the major breast cancer subtypes [13]. Already some attempts to conduct proteogenomic characterization of human cancers are resulting in improved characterization of candidate driver genes and genomic abnormalities [47]. Alternative clustering approaches, performing joint inference from distinct assays and generating a single integrated cluster assignment, were equally useful in characterizing breast cancer and glioblastoma subtypes starting from genome and transcriptome data [30, 94]. Other attempts integrated molecular profiles with previous knowledge captured in gene interaction networks to stratify ovarian, uterine and lung cancer patients [95]. Multi-assay biomarker discovery relies on integrative regression approaches, which differ from one another in the use of data of different types to construct the predictor [96, 97], and on network analysis approaches, which have so far included correlation- and mutual information-based networks [98], Bayesian and graphical models [99]. With the growing interest in approaches that combine primary tumor data such as transcriptome profiles with networks and pathways, several initiatives provide tools to objectively evaluate the improvement in outcome prediction performances [100]. Although the outlined techniques are fast evolving, their widespread use beyond a few worldwide projects has so far been hindered by the relatively low availability of omic data sets involving more than one data type measured in the same set of cancers, the theoretical and computational hardship of analytical frameworks allowing joint inference from multi-assay data, the high dimensionality of high-throughput multi-assay data and the incomplete annotation and partial compatibility of different genomic features.

## Conclusions

The past 15 years have seen an amazing growth in the ability to generate molecular data at multiple complementary levels of gene expression, therefore enabling the generation of molecular portraits in unprecedented detail in a variety of cancer contexts. However, with a few exceptions, these investments have failed to substantially improve diagnosis, prognosis or treatment of cancer. A fundamental reason for this discrepancy between data generation and clinical improvement is the relatively immature use of experimental designs and analytical methodologies to handle and meaningfully interpret high-throughput data types. The majority of attempts to stratify tumors or to predict clinical outcome to date have used transcriptome-based data without consideration of the effects in protein levels or have postulated linear correlation of data across these levels. Future advances will focus on increasing the information content of each data type, reducing the amount of input biological material, getting relevant data in less analysis time and improving mathematical modeling to uncover relationships between diverse and complementary data types and clinical phenotypes. Continuous attempts to fully describe the functional alterations at the multiple levels of gene regulation in cancer will be instrumental to the effective interpretation of cancer molecular data and to their translation into the clinic.

**Key Points**

- Transcriptome profiling has resulted in limited clinical utility despite tremendous efforts.
- The potential prognostic and predictive value of profiling protein levels of gene regulation is pitifully unexplored and deserves careful assessment.
- Multiple types of ‘omic’ data provide complementary information of which integration might be crucial to improve the assessment of tumor prognosis.
- Effective feature selection and mathematical modeling strategies should be developed in the analysis of individual data types and in the integration thereof.

## Funding

Biotechnology Program of the University of Trento.
